# Syphilis-Associated Proteinuria and Hepatitis in the Setting of Human Immunodeficiency Virus (HIV) Co-Infection

**DOI:** 10.1155/2022/7247946

**Published:** 2022-09-22

**Authors:** Hanish Jain, Garima Singh, Elizabeth Asiago-Reddy

**Affiliations:** ^1^Department of Medicine, Division of Infectious Diseases, State University of New York, Upstate Medical University, Syracuse, NY, USA; ^2^Department of Medicine, Saint Vincent Hospital, Worcester, MA, USA

## Abstract

Syphilis has long been known as “the great imitator”, mimicking a wide variety of diseases, and often its diagnosis is delayed or missed. It remains an important public health issue that continues to occur at high rates among patients with HIV. We report a case of a 52-year-old man who presented with a constellation of unusual symptoms highlighting that syphilis should be included in the differential diagnosis in patients with HIV presenting with abnormal liver enzymes, rash, proteinuria, conjunctivitis, and/or sexual risk factors.

## 1. Introduction

The rate of syphilis continues to rise in the United States, despite effective antitreponemal therapy, essentially due to the increasing incidence of primary and secondary syphilis among men who have sex with men (MSM) [1]. Clinical manifestations of syphilis can vary greatly because it can involve almost every organ. The variability in the clinical presentation seen in syphilis can make the diagnosis challenging. Rapid diagnosis and treatment of syphilis as well as rapid identification and treatment of sexual contacts are needed to reverse the trend of increasing incidence [2]. It is important to consider secondary syphilis in the differential diagnosis in patients with HIV presenting with systemic disease.

## 2. Case Presentation

**Figure 1:**
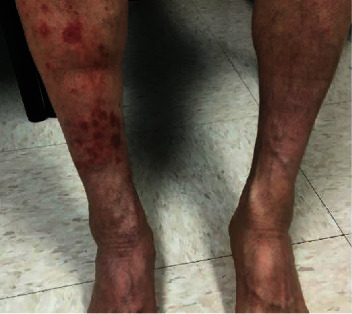
Nickel and dime lesion on the right leg.

A 52 year-old man (MSM) with a history of Gilbert's disease, HIV for 18 years well controlled on emtricitabine-rilpivirine-tenofovir alafenamide (Odefsey) presented to his primary care physician with a constellation of unusual symptoms of low-grade fevers, fatigue, muscle aches, joint stiffness, bilateral red eyes with tearing, rash, and dark urine for 2 months. The patient stated he initially noticed low-grade fevers followed by fatigue, muscle aches, and joint stiffness that responded to as-needed pain medications. He scheduled an urgent visit when he started noticing bilateral red eyes with tearing but no visual disturbances, a rash that started on his right leg with no associated pain or itching, and dark urine gradually worsening over the last week. The patient had no other significant medical history, alcohol intake, illicit drug use, herbal supplement intake, or family history of autoimmune disorders. The patient worked outside, often had contact with ticks, and frequently picked ticks off his body when he was out in the woods. The patient did not recall having a bull eye rash at the time. The patient was sexually active and did have unprotected sex with a single partner since the last sexually transmitted infections screen 3 months ago. The patient was otherwise healthy. On examination, the patient was hemodynamically stable, afebrile, and appeared well, with a nickel and dime lesion on his right leg (Figure 1). His blood tests showed elevated liver biochemical tests with alanine aminotransferase (ALT) 128 U/L, aspartate aminotransferase (AST) 68 U/L, alkaline phosphatase 1193 U/L, total bilirubin 2.7 mg/dL, erythrocyte sedimentation rate 114, c-reactive protein 28.7, urine analysis with protein >500. HIV viral load remained undetectable and CD4 count was normal. Lyme antibodies came back negative. Syphilis IgG/IgM screen resulted reactive with rapid plasma reagin (RPR) reactive titer of 256. The patient was recommended to have an admission for lumbar puncture and intravenous penicillin but declined and instead preferred treatment with weekly penicillin *g* benzathine injections *x* 3 doses. The patient had significant improvement in his symptoms and labs normalized after 1 month. RPR Titer came back at 16 after 3 months.

## 3. Discussion

The incidence of syphilis among patients with HIV, especially MSM, has been rising in the United States, and several outbreaks have been reported [1]. Syphilis most commonly manifests as a genital ulcerative disease during the primary stage and as rash, fever, and adenopathy during the secondary stage. Dissemination of treponemes throughout the body occurs during the secondary stage, affecting both mucocutaneous and visceral sites [3]. Secondary syphilis is typically a systemic disease. The patient often presents with a variety of symptoms, such as malaise, sore throat, headache, weight loss, low-grade fever, pruritus, muscle aches, and dermatologic manifestations like the “nickel and dime” maculopapular rash on the body not sparing the palms or soles. Lymph node enlargement is present in the great majority of patients. Although liver involvement due to syphilis has been recognized in the recent literature, published reports are sparse, with the largest series consisting of only 32 cases [3]. Hepatitis represents a frequently unknown and unusual manifestation of early syphilis syndrome. Hepatic involvement has been classically reported as a disproportionally elevated alkaline phosphatase level owing to pericholangiolar inflammation in the setting of secondary syphilis [4]. Patients may also present with elevated AST and ALT levels and an elevated total bilirubin level [5]. Blood tests may also reveal renal involvement, most often seen as isolated proteinuria although glomerulonephritis or nephrotic syndrome can also occur in secondary syphilis [6]. Ocular manifestations include iritis, anterior uveitis, arthritis, and conjunctivitis. Circulating immune complexes containing treponemal antigen and human fibronectin, together with antibodies and complement, are present in this stage of infection. Their deposition in relevant organs is thought to play a role in the pathogenesis of these syndromes [7]. Secondary infection may be more aggressive and there is an increased rate of early neurological and ophthalmic involvement [8]. Even though our patient declined a lumbar puncture it should be offered to all patients to rule out neurological involvement. Collectively, the treponemal and nontreponemal serologic laboratory blood tests are referred to as serologic tests for syphilis. Diagnosis is generally made with the detection of treponemal IgM/IgG antibodies as an initial screening test followed by automated RPR [9]. Although the clinician should be aware of the potential for false-negative serology in both primary and less commonly secondary syphilis. All patients with HIV should be treated with a penicillin-based regimen that is adequate for the treatment of neurosyphilis. In the case of penicillin allergy, data indicate that ceftriaxone 1-2 g daily either IM or IV for 10–14 days can be used as an alternative treatment [10]. Relapse of infection is more likely in patients with HIV, and careful follow-up is required [8]. The most important elements in the effective management of syphilis are frequent screening, treating patients with syphilis quickly, identifying and treating sexual contacts, and rescreening.

To conclude, this case emphasizes the importance of early recognition of rare presenting characteristics of syphilis and the importance of a high index of suspicion, especially when evaluating proteinuria, glomerulonephritis, hepatitis, rash, or conjunctivitis in patients with HIV.
